# Novel frontier in wildlife monitoring: Identification of small rodent species from fecal pellets using near‐infrared reflectance spectroscopy (NIRS)

**DOI:** 10.1002/ece3.9857

**Published:** 2023-03-19

**Authors:** Maria W. Tuomi, Francisco J. A. Murguzur, Katrine S. Hoset, Eeva M. Soininen, Eero J. Vesterinen, Tove Aa. Utsi, Sissel Kaino, Kari Anne Bråthen

**Affiliations:** ^1^ Department of Arctic and Marine Biology UiT The Arctic University of Norway Tromsø Norway; ^2^ Section of Ecology Department of Biology University of Turku Turku Finland; ^3^ Department of Ecology Swedish University of Agricultural Sciences Uppsala Sweden; ^4^ Biodiversity Unit University of Turku Turku Finland; ^5^ Department of Agricultural Sciences University of Helsinki Helsinki Finland; ^6^ Department of Arctic and Marine Biology UiT The Arctic University of Norway Alta Norway

**Keywords:** abundance index, diet, DNA metabarcoding, field method, multispecies community, noninvasive sampling, tundra

## Abstract

Small rodents are prevalent and functionally important across the world's biomes, making their monitoring salient for ecosystem management, conservation, forestry, and agriculture. There is a growing need for cost‐effective and noninvasive methods for large‐scale, intensive sampling. Fecal pellet counts readily provide relative abundance indices, and given suitable analytical methods, feces could also allow for the determination of multiple ecological and physiological variables, including community composition. In this context, we developed calibration models for rodent taxonomic determination using fecal near‐infrared reflectance spectroscopy (fNIRS). Our results demonstrate fNIRS as an accurate and robust method for predicting genus and species identity of five coexisting subarctic microtine rodent species. We show that sample exposure to weathering increases the method's accuracy, indicating its suitability for samples collected from the field. Diet was not a major determinant of species prediction accuracy in our samples, as diet exhibited large variation and overlap between species. fNIRS could also be applied across regions, as calibration models including samples from two regions provided a good prediction accuracy for both regions. We show fNIRS as a fast and cost‐efficient high‐throughput method for rodent taxonomic determination, with the potential for cross‐regional calibrations and the use on field‐collected samples. Importantly, appeal lies in the versatility of fNIRS. In addition to rodent population censuses, fNIRS can provide information on demography, fecal nutrients, stress hormones, and even disease. Given the development of such calibration models, fNIRS analytics could complement novel genetic methods and greatly support ecosystem‐ and interaction‐based approaches to monitoring.

## Graphical abstract

Calibration models based on near‐infrared reflectance spectra (fNIRS) of single fecal pellets can accurately predict taxonomic identity of five co‐existing microtine rodent species. The method is cost‐effective and non‐invasive, and based on our results, the models accurately classify weathered feces and samples from different regions.

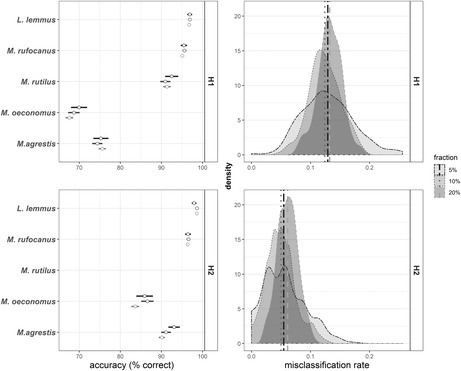

## 1 INTRODUCTION

Small rodents are prevalent in ecosystems across the globe, with many species acting as ecosystem engineers (Dickman, 1999) or keystone species (Ims & Fuglei, 2005); with numerous rare species of high conservation value (Green et al., 2013) and many invasive populations with profound effects on ecosystem functioning (Dickman, 1999). Monitoring and research of small rodents is thus a globally salient enterprise for ecosystem management, conservation, forestry, and agriculture. Yet, obtaining population‐ or community‐level data on small rodents is often challenging, as these small and cryptic animals are elusive (Green et al., 2013) and cost‐effective methods for large‐scale sampling of their multispecies communities are largely missing (Engeman & Whisson, 2006; Heisler et al., 2016). Estimation of small rodent community composition, population size, and density relies on a variety of trapping, sampling, and indexing efforts, for example, pitfalls, live‐, snap‐, hair‐, or camera traps and systematic incidental observations (Engeman & Whisson, 2006; Fauteux et al., 2018; Soininen, Jensvoll, et al., 2015), or on counts of burrows, runways, winter nests, owl pellet contents, and feces (Green et al., 2013; Heisler et al., 2016). Trapping methods, while providing actual estimates on species identity, abundance, and key demographic parameters, are laborsome (Engeman & Whisson, 2006; Villette et al., 2016) and often inadequate for high‐intensity sampling with large spatial or temporal coverage (Heisler et al., 2016; Villette et al., 2016).

Multitude of wildlife censuses rely on feces counts (Campbell et al., 2004; Green et al., 2013; Karels et al., 2004; Kohn & Wayne, 1997), as feces are a noninvasive source of readily available samples. Acquiring feces does not require being in contact with the animal, thus decreasing the risk of infections and animal stress, and feces can provide information from seasons and times when researchers are not present (Kohn & Wayne, 1997; Whisson et al., 2005). Feces are organic material whose structural and chemical properties are determined by the mechanical, biochemical, and microbiological processing of ingested biomass throughout the digestive pathway. Feces—and the chemical and genetic information they store—could therefore allow for the determination of diverse ecological and physiological variables indicative of species interactions (c.f. Ehrich et al., 2019; Vance et al., 2016), if combined with suitable molecular, endocrinological, or spectral methods (Galan et al., 2012; Schwarzenberger, 2007; Zemanova, 2021). Indeed, realizing the potential of fecal data for large‐scale ecological monitoring calls for a set of cost‐effective high‐throughput analytical methods (Vance et al., 2016; Zemanova, 2021). Such methods, if embedded in appropriate monitoring schemes (Yoccoz et al., 2001), could provide access to questions pertinent to ongoing biodiversity shifts and ecosystem changes (Lenoir & Svenning, 2015; Pecl et al., 2017; Wintle et al., 2010). To be feasible and applicable, these methods should be robust and sensitive across space and time, utilize easily obtainable samples, be quick in the preprocessing of the samples, and be cheap and fast (Engeman & Whisson, 2006; Whisson et al., 2005). Analytical methods that are nondestructive for the fecal samples themselves increase their utility by allowing subsequent application of further analytical methods. For instance, rapid advances in noninvasive and nondestructive genetic methods allow for increasingly cost‐effective taxonomic analysis of feces (Zemanova, 2021), given sufficiently preserved genetic material.

Alongside genetic methods, near‐infrared reflectance spectroscopy (NIRS) is a promising and highly versatile method for diverse ecological monitoring (Vance et al., 2016). NIRS is widely applied in agriculture and petrochemical industry (Pasquini, 2003), with increasing representation in different fields of ecology (Aw & Ballard, 2019; Murguzur et al., 2019; Vance et al., 2016; Villamuelas et al., 2017). NIRS is a rapid and nondestructive spectral analytic tool for assessing quantitative and qualitative variables based on the physicochemical information stored in the NIR spectra, including, for example, taxonomic or demographic information and fecal chemistry, or even stress or disease (Vance et al., 2016). After initial investment, the method is very affordable. Sample preprocessing is minimal, mainly involving sample material homogenization and drying (Pasquini, 2003). The scanning procedure of a sample takes only seconds and does not require specialized personnel or laboratories. Once a calibration model for the variable of interest (e.g., species identity) is validated, scanning and analysis of any number of further samples comes with no extra cost of, for example, reagents or genetic primers. After scanning, samples can be analyzed with other methods, and the existing spectra can be later analyzed for new variables using respective calibrations. Fecal NIRS (fNIRS) has been used to predict species identity, demographic parameters (Aw & Ballard, 2019; Tolleson et al., 2005; Wiedower et al., 2012) and diet quality (Foley et al., 1998; Villamuelas et al., 2017) of several wild animals from large mammals to insects (Vance et al., 2016). However, the use of fNIRS in wildlife research and monitoring remains rather unknown to the ecologist community, in comparison with the widespread use of remote sensing (Kerr & Ostrovsky, 2003; Pettorelli et al., 2014) and high‐throughput genetic barcoding (Yoccoz, 2012).

As with any analytical method, there are potential caveats to using fNIRS for small rodent monitoring. First, as feces are collected from the field, they are exposed to ambient weather. This can compromise prediction accuracy because leaching and microbiological processes can change pellet chemical composition (Jenks et al., 1990; Kamler et al., 2003), similar to issues linked with degrading DNA. Second, variation between animal individuals may affect their fecal composition and NIR spectra and hence decrease or confound prediction accuracy of species. In particular, diet quality affects fNIR spectra (Stuth et al., 2003; Villamuelas et al., 2017) and may compromise prediction accuracy whenever considerable dietary overlap between species occurs. Especially for coexisting and competing generalist rodent species, the effect of diet is likely to be complex, as diets overlap and are dependent on, for example, season and forage item availability (Soininen et al., 2013). In addition, sex and reproductive status may affect fNIR spectra (Tolleson et al., 2005), increasing variation in the spectral data. Third, if factors driving the species‐signal in fNIR spectra differ between populations, fNIRS calibrations that cover only spatially limited target populations can yield unreliable results on samples from a different population; an issue avoided by genetic methods of well‐preserved samples. However, recent studies presented NIRS calibrations applicable across species and geographic regions (Murguzur et al., 2019; Villamuelas et al., 2017), revealing the potential for cross‐regional or global NIRS and fNIRS calibrations.

Here, we develop fNIRS calibration models for rodent taxonomic determination from single pellets (>0.025 g of dry weight). Our main hypothesis is that fecal properties differ between taxa, allowing for classification of individuals to genus and species based on their fNIR spectra (H1 and H2). However, this separation capability might erode with the effect of exposure (H1 vs. H2) due to leaching, irradiation, and decomposition. Prediction accuracy of individual samples may also be linked with diet composition (H3), which we tested by comparing fNIRS data to DNA metabarcoding data of the same pellets. Additionally, spectra may display regional differences between populations (H4). For the latter, we contrast two subhypotheses: H4.1 and H4.2 that calibration models based on samples from one region may perform poorly with samples from other region and H4.3 that a calibration model including all regions successfully classifies independent test individuals from all regions. Finally, we demonstrate how our calibration model and the modeling framework would be used in practice to predict species identity of new fecal samples.

## 2 MATERIALS AND METHODS

**FIGURE 1:**
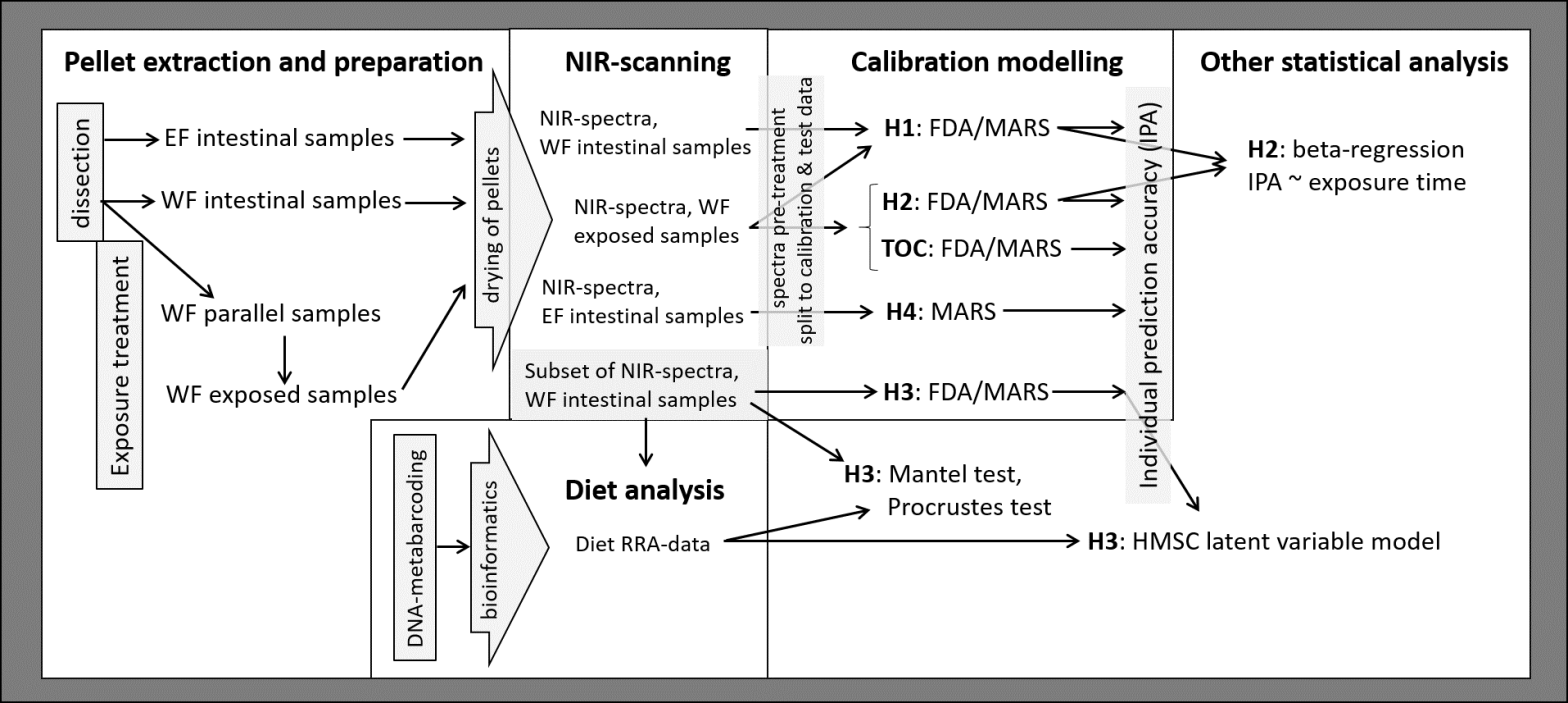
Illustration of the analytical workflow. Detailed description of calibration models is presented in Table 1.

We outline the workflow of building NIRS calibrations and testing our hypothesis in Figure 1. We detail the following steps: rodent fecal sample extraction, sample processing and experimental treatment, NIRS scanning and spectra pretreatment, calibration modeling including model building, validation and testing, diet molecular analysis including DNA metabarcoding, bioinformatics and modeling potential confounding of diet, as well as regression modeling of NIRS calibration model results. Further details on molecular methods and calibration modeling are provided in the Supplementary material S1

### 2.1 Rodent data and fecal samples

We collected fecal samples from animals trapped as part of a long‐term rodent population monitoring time series. The set of rodent individuals included in the calibration modeling incorporated substantial variation in environmental conditions of their trapping site, season, and year, as well as variation in individual physiological condition related to reproductive status and age (Table S1a). The largest set of individual fecal samples (*n* = 472) came from West Finnmark, Northern Norway, encompassing two research areas ca. 55 km apart (hereafter West Finnmark or “WF”)—Joatka research area (JRA; 69°45’N, 23°55’E) and Skillefjordnes (Skirvinjárga; 77°86’N, 05°86’E). Small rodent guild at WF includes five out of seven small rodent species occurring throughout Northern Fennoscandia: Norwegian lemmings (*Lemmus lemmus*), gray‐sided voles (*Myodes rufocanus*), red voles (*Myodes rutilus*), root voles (*Microtus oeconomus*), and field voles (*Microtus agrestis*). JRA has in total 77 trapping quadrats of 15 by 15 m, including snowbed, heath, meadow, and wetland habitats. Trapping was conducted biannually, during early (mid‐June) and late (early‐mid September) growing season. Samples were included from a full population cycle between 2010 and 2014, with the largest rodent peak in 40 yr occurring in 2011–2012 (see Ekerholm et al., 2001; Hoset et al., 2014 for details on index trapping protocol). As the number of *M. agrestis* individuals from JRA was not sufficient, we supplemented the JRA samples with individuals (*n* = 40) trapped in comparable heath, meadow, and snowbed habitats at Skillefjordnes (see Ruffino et al., 2015 for site description). We also included three *M. rufocanus* individuals from this site. Altogether, WF fecal samples included 107 *L. lemmus*, 72 *Myodes rufocanus*, 103 *M. rutilus*, 93 *Microtus oeconomus*, and 97 *M. agrestis* (Table S1a).

Another set of fecal samples (*n* = 85) came from animals trapped in three river catchments in East Finnmark—Ifjordfjellet (70° N, 27° E), Komagdalen and Vestre Jakobselv (70–71° N, 28–31° E; hereafter East Finnmark or “EF”). Similarly to WF, trapping was conducted biannually in 15 by 15 m quadrats, but samples were only collected in 2015. A total of 80 quadrats are distributed in heath and meadow habitats. The EF samples included mainly *Microtus oeconomus* (*n* = 42) and *Myodes rufocanus* (*n* = 38), with two *L. lemmus* and one *Myodes rutilus* (Table S1a).

All rodents were frozen after trapping, and information of trapping date and sampling quadrant was recorded. We also recorded individual rodent body mass, sex, species identity, and female reproductive status (visible pregnancy). Dataset included individuals of varying age based on the wide body mass distribution (*L. lemmus* 32–106 g, *M. rufocanus* 8–65 g, *M. rutilus* 12–36 g*, M. oeconomus* 22–90 g, and *M. agrestis* 17–66 g).

Feces were carefully sampled from the intestine without damaging or breaking the intestinal tissue, avoiding contamination with blood or other compounds that could affect the NIR spectra. From each individual from WF, we collected consecutive pellets in two Eppendorf tubes, to form two parallel samples, while of EF samples no parallel samples were taken. All pellets were dried at 40°C for minimum of 48 h or until dry and stored in Eppendorf tubes in room temperature. One parallel WF sample and all EF samples were NIR‐scanned without exposure treatment (intestinal samples), while the other WF parallel sample was scanned after the exposure treatment (exposed samples).

### 2.2 Exposure treatment

We subjected one set of parallel WF samples to ambient weather conditions (hereafter “exposure treatment”) in Tromsø, Northern Norway, during autumn 2014 to incorporate the effects of leaching and bleaching due to UV radiation on NIR spectra in the calibration model. Only four species were included in the weathering treatment, as *Myodes rutilus* pellets were not suited for weathering due to a frequently liquid consistency and small size of the pellets. We placed the pellets on wooden frames of 50x50 cm, with a 1 x 1 mm nylon mesh in the bottom. Nylon mesh was used as a bottom material because it does not release chemicals to the feces and allows for water drainage and evaporation. Each frame had pellets of only one species to avoid contamination. Each frame had ca. 150 pellets. For *L. lemmus* and *Myodes rufocanus* pellets, we used two frames per species, whereas *Microtus oeconomus* and *M. agrestis* had one frame each. Frames were placed on heath vegetation in September, and weather conditions during the treatment were variable, including rain, sunshine, frost, and temperatures both above and below zero. During 6 weeks, we weekly took ca. 25 pellets for scanning from each frame.

### 2.3 NIRS scanning of the fecal pellets

We scanned individual intestinal and exposed samples without further sample prepreparation. Due to the small size, we used a custom‐built, Ø4mm sample holder and pressed the pellet flat with an Ø4mm metal rod in order to prevent light from penetrating through the sample. A few samples were too small to cover the whole area, but they did not appear as outliers during the modeling process

NIR spectra were collected using a FieldSpec 3 (ASD Inc., Boulder, Colorado, USA): each spectrum was recorded as reflectance using monochromatic radiation at 1.4‐nm intervals in the 350–1050 nm range and 2 nm intervals in the 1050–2500 nm range, and interpolated to 1 nm resolution. We scanned each sample three times by rotating the sample holder between scans in order to account for angular effects on light scattering. We used the mean of the three spectra in the further analysis. We used the R package *prospectr* (Stevens & Ramirez‐Lopez, 2015) for spectra preprocessing, that is, applying splice correction and normalizing the spectra. The spectral regions in the 350–399 nm and 2451–2500 nm ranges were removed from the dataset due to instrumental noise

### 2.4 DNA‐metabarcoding of rodent feces

After scanning, we analyzed a subset of intestinal WF samples (*n* = 385) for diet species composition with DNA metabarcoding. All wet laboratory work was performed by Center of Evolutionary Applications, University of Turku, Finland. We used a DNA metabarcoding approach similar to Soininen, Gauthier, et al. (2015). We here give a summary of the methods, and a detailed description is included in supplementary materials S1 (p. 2: Detailed DNA‐metabarcoring of rodent feces). DNA was first extracted and thereafter amplified using universal plant primers “g‐h” and “c‐h” that target chloroplast trnL intron (Taberlet et al., 1991, 2007). Final DNA libraries were constructed in the second PCR (see, e.g., Vesterinen et al., 2018). Resulting DNA libraries were purified and size‐selected using an SPRI bead clean‐up (see Vesterinen et al., 2016), and concentrations were measured by Qubit (Invitrogen; www.invitrogen.com) and finally pooled in equimolar quantities. Sequencing was done on 316 v2 and 318 v2 chips with Ion PGM (Life Technologies, manual cat nr 00009816, Rev C.0).

The four sequencing runs yielded altogether 12,575,869 quality‐controlled reads that could be assigned to original samples. The reads were uploaded to CSC servers (IT Center for Science, www.csc.fi) for trimming and further analysis. Bioinformatic steps were carried out following Schmidt et al. (2018) with several modifications (p. 3–4: Bioinformatics). To summarize, reads were merged, filtered for quality, cut for primers, collapsed into unique haplotypes, and finally clustered into zero‐radius OTUs using softwares *usearch* (Edgar, 2010) and *cutadapt* (Martin, 2011). We were able to map 7,726,911 reads (~93% of the trimmed reads) to our original samples. The *trnl* OTUs were initially identified to taxa using the *usearch* “*sintax*” classifier with a database consisting of artic plant trnl sequences (Willerslev et al., 2014) using 70% probability threshold for taxonomic assignation. After this, read counts for each diet taxon in each sample were transformed to relative read abundances (RRA), as read abundances may actually be less misleading than presence/absence conversions (Deagle et al., 2018).

### 2.5 Calibration modeling of genus and species identity

After obtaining NIR spectra and diet data on individual samples, we built calibration models for genus and species identification and further used regression, multivariate, and latent variable modeling in order to address our four hypotheses and test of concept (Figure 1, Table 1). To build calibration models, we applied multivariate adaptive regression splines (MARS; Friedman, 1991) both directly and in a flexible discriminant analysis framework *(FDA;* Hastie et al., 1994 *; see* Table 1 *)*. For this, we used R packages *earth* (Milborrow, 2014) and *mda (*Hastie et al., 2015 *)*. We chose this approach as it deals well with high‐dimensional input data and allows for additive effects and interactions between variables (Friedman & Roosen, 1995) and assumes nonlinear responses or decision boundaries (Hastie et al., 1994). A more detailed description of the modeling and a comparison with other common chemometric approaches is provided in Supplementary material S1 (p. 4, Calibration modeling of genus and species identity). We used R version 3.5.1 (R Core Team, 2018) for all statistical analyses and the *ggplot2* (Wickham, 2009) environment for plotting all figures.

**TABLE 1:** Summary of calibration model methodology. Column « **Hypothesis »** refers to the tested question.

Hypothesis	Model	Response variable	Calibration data	Test data
*H1*	FDA/MARS, degree = 2 no. of models = 2	**genus** (Lemmus, Myodes, Microtus); **species** (Llem, Mruf, Mrut, Moec, Magr)	WF intestinal and exposed samples^a^	WF intestinal and exposed samples^a^
*H2*	FDA/MARS, degree = 3 no. of models = 2	**genus** (Lemmus, Myodes, Microtus); **species** (Llem, Mruf, Moec, Magr)	WF exposed samples^a^	WF exposed samples^a^
*H3*	FDA/MARS, degree = 3 no. of models = 1	**species** (Llem, Mruf, Mrut, Moec, Magr)	WF intestinal samples^b^	WF intestinal samples^a^
*H4*	MARS, degree = 3 no. of models = 3	**Mruf, Moec**	H4.1a = WF^a^ H4.1b = EF^b^ H4.2 = WF & EF^c^	H4.1a = 100% EF, WF^a^ H4.1b = 100% WF, EF^b^ H4.2 = WF & EF^c^

*Note*: Column « **Model»** refers to the used model type, that is, FDA models applying MARS (FDA/MARS) and direct application of MARS. Degree indicates 1‐way (degree = 2) or 2‐way (degree = 3) interactions of model variables. Number of models indicates that each response variable, for example, genus or species, has its own calibration model. Column « **Response variable »** describes the categories predicted by the calibration model. Column « **Calibration data»** and « **Test data** » describe the datasets used to calibrate the model and test its generalization error. Total number of rdMCCV iterations per model is 600, that is, the product of number of MCCV iterations (*n* = 200) and number of data splits (*n* = 3).

Abbreviations: EF, East Finnmark; Llem, *Lemmus lemmus*; Magr, *M. agrestis*; Moec, *Microtus oeconomus*; Mruf, *Myodes rufocanus*; Mrut, *M. rutilus*; WF, West Finnmark.

^a^ 95%/5%, 90%/10%, and 80%/20% of each species and exposure week.

^b^ 95%/5%, 90%/10%, and 80%/20% of each species.

^c^ 95%/5%, 90%/10%, and 80%/20% of each species, exposure week, and region.

We built separate calibration models for genus and species, and to test each hypothesis (H1‐H4, Table 1). Usually, in a real‐life application, only one model would be applied to the entire calibration dataset, as shown in Box 1. However, for the purpose of rigorous hypothesis testing, here we evaluated the models using a modified, repeated double Monte Carlo cross‐validation *(*Filzmoser et al., 2009 *; rdMCCV;* cf. Xu et al., 2004 *)* with 200 iterations. Within each iteration, we divided the modeling dataset into a *calibration set* dataset used for model fitting and variable selection and an *MCCV test set* for an assessment of model prediction accuracy, that is, of the generalization error of each iterated calibration model *(*Filzmoser et al., 2009 *; reported as model misclassification rate and species prediction accuracy;* Pasquini, 2003 *)*. The purpose of applying rdMCCV was to estimate how model performance and misclassification rates vary across sets of calibration data (Filzmoser et al., 2009) and to estimate error rates for individual samples *(*cf. Liu et al., 2008 *)*. Furthermore, we repeated the rdMCCV procedure with 95%/5%, 90%/10%, and 80%/20% data splits between calibration and test sets. This increased the number of total rdMCCV iterations per model to 600 (Table 1). To ensure the allocation of targeted variability between calibration and test sets, the data were split (depending on the model) as fixed percentage of each species; species and exposure treatment duration; or species, exposure treatment duration, and region (Table 1). We used MARS algorithms with one‐way or two‐way interactions of model variables, that is, hinge functions of NIR spectra (Table 1), depending on which interaction structure provided the lowest misclassification rate of test samples based on preliminary models with 20 iterations (Filzmoser et al., 2009)

To test hypothesis H1 (*Can we predict genus or species identity based on their fNIR spectra?*), we built calibration models using WF intestinal and exposure sample spectra (Table 1) and constructed separate models for species and genus‐level identification. Model response variable had three levels in the genus model and five levels in the species model (Table 1). In our dataset, the genus level *Lemmus* consists of only one species. In spite of this, we included the taxon in both species and genus‐level models, to systematically assess model predictive performances. To ensure comparability with H2 model (see below), we ran an additional rdMCCV calibration model without *Myodes rutilus*. This did not change results for the other four species, and results are not included.

To test hypothesis H2 (*Does feces exposure reduce prediction accuracy?*), we built calibration models for genus and species identity with data from the exposure treatment only. Modeling follows the description for H1, with deviations described in Table 1. We excluded *Myodes rutilus*, as the species was omitted from the exposure treatment. We then tested whether individual sample prediction accuracy was explained by exposure time. For this, we used Bayesian regression models on species identity results of H1 and H2 models separately for each species and model. As response variable, we used the *individual sample prediction accuracy* (hereafter IPA). To calculate this, we extracted the results per each individual in each model iteration, with “0” denoting a misclassification, and “1” a correct classification. We then averaged classification result across all iterated model runs. Prior to analysis, we transformed the IPA values as

(1) $${\mathrm{IPA}}_{\text{beta}}=\frac{\mathrm{IPA}\left(n-1\right)+0.5}{n}.$$

to avoid zero–one inflated data (Smithson & Verkuilen, 2006). As predictor variable, we used exposure treatment time (number of weeks from 0 to 6 as a numerical fixed factor). Due to the beta‐distribution (0 < y < 1) of IPA values, we fitted beta‐regressions with a logit‐link using package *rstanarm* and with the function's default weakly informative priors *(*Muth et al., 2018 *)*. We fitted the model using Markov chain Monte Carlo sampling (MCMC) with 4 chains and 2000 iterations in each chain (rstanarm default; Muth et al., 2018). We checked sampling quality of draws from the target posterior distribution for MCMC via numerical checks (R̂ <1.1 and n_eff_ > 1200 for all model parameters) and visually with trace plots, and we inspected and confirmed good model fits by visual posterior predictive checking (Muth et al., 2018) with package *bayesplot* (Gabry & Mahr, 2019).

To test of hypothesis H3 (*Does diet confound prediction accuracy?*), we based our inference jointly on two different modeling strategies. First, we assessed the correlation between fNIR and diet RRA multivariate datasets using Mantel test with Bray–Curtis distance and Procrustes test (Jackson, 1995) using package *vegan* (Oksanen et al., 2018). While Mantel test is widely used, it may fail to account for the mean–variance relationship of the diet data *(*Warton et al., 2012 *)*; hence, we used the Procrustes test in addition. Second, we visually explored whether highly misclassified individuals (IPA < 0.60) displayed deviant diet composition compared with correctly classified individuals of the same species. The IPA values used here were derived from calibration models built as described for H1 but including only those intestinal samples used for DNA metabarcoding (*n* = 380; Table 1). To discern patterns of intra and interspecific diet similarity, we applied multivariate latent variable modeling (model‐based unconstrained ordination; Warton et al., 2015) on diet RRA data. We fitted the model using Bayesian Markov Chain Monte Carlo estimation within the HMSC modeling framework (package *Hmsc*, Blanchet, 2013; Ovaskainen et al., 2017). To estimate the model (latent) parameters, we used two MCMC chains each with 15,000 iterations with a burn‐in of 5000 and model default priors (see Ovaskainen et al., 2017). MCMC sampling quality of posterior draws was checked numerically and was deemed good (R̂ <1.1 and n_eff_ > 1750). We then plotted the 2D latent variable plot of diet composition and added the information of sample IPA values in the plot

To test hypothesis H4 (*Are models generalizable between regions?*), we used *Microtus oeconomus* and *Myodes rufocanus* data from both WF and EF, as these were the only numerous species in EF. First (H4.1 and H4.2), we built separate calibration models based on data from either WF or EF only and predicted species identity of test data including both regions (see Table 1). Thereafter (H4.3), we built calibration models with WF and EF data and predicted MCCV test set individuals from both regions

Finally, we demonstrate the practical application of our calibration model and the rdMCCV framework. This includes building a calibration model (Step 1), predicting new samples not seen by the calibration model (Step 2) and visualizing the calibration model predictions and misclassification risk of WF and EF samples in the FDA‐model discriminant space (Step 3). Methods and results for each step are described in Box 1.

## 3 RESULTS

### 3.1 H1: Prediction accuracy of genus and species identity

**FIGURE 2:**
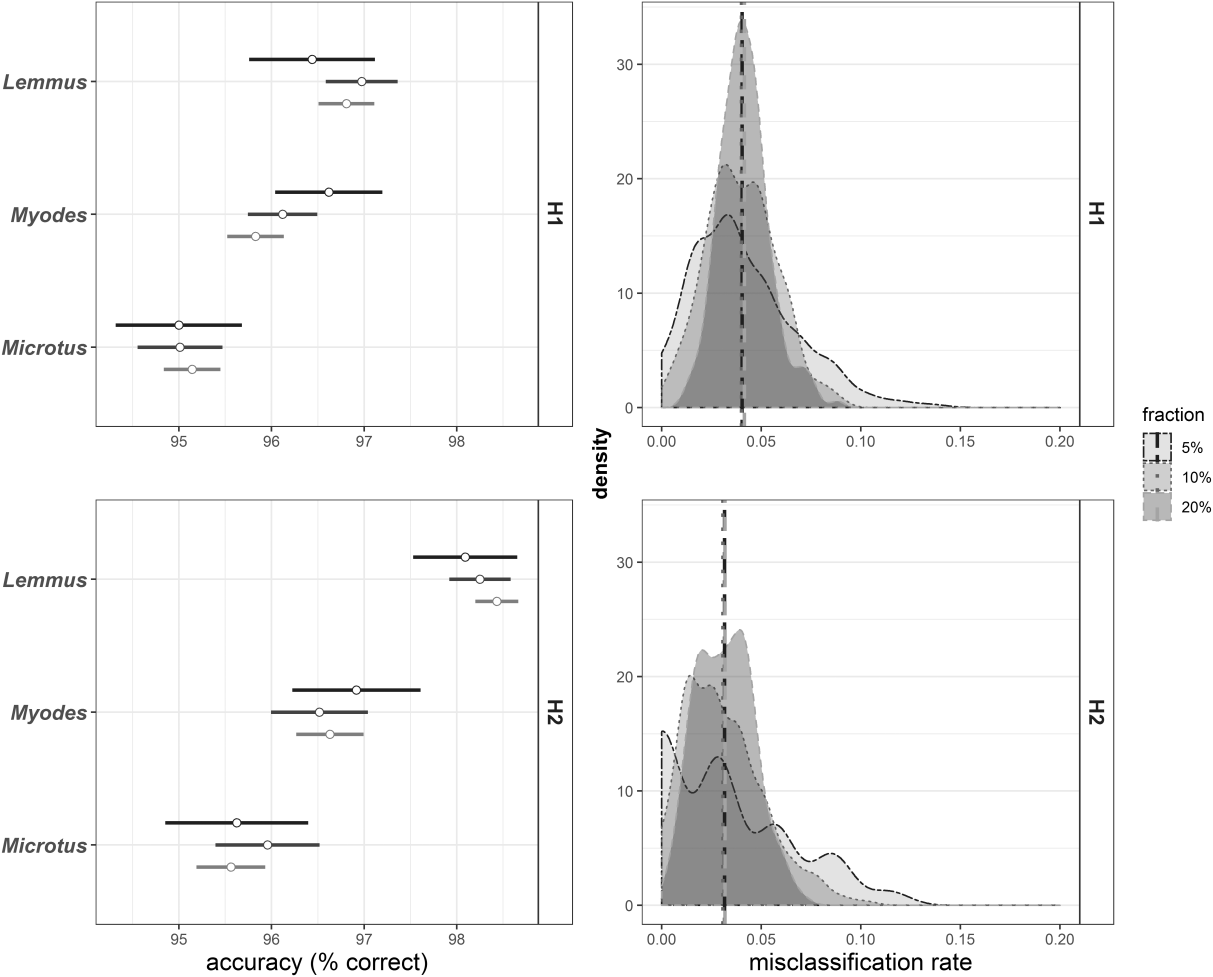
Predictive ability of genus‐specific fNIRS calibrations for small rodents from Finnmark, Norway. **Left panels:** prediction accuracy of each genus (mean ± sd) separately. **Right panels:** density plots of model misclassification rates. **Upper row** shows results from calibration models used for H1 (i.e., including all data from West Finnmark), **lower row** for H2 (i.e., including only samples used to test exposure effect). Results are divided by data splits (fractions) with 200 iterations each, where 5%, 10%, and 20% of data were randomly assigned as validation data (using rdMCCV framework).

**FIGURE 3:**
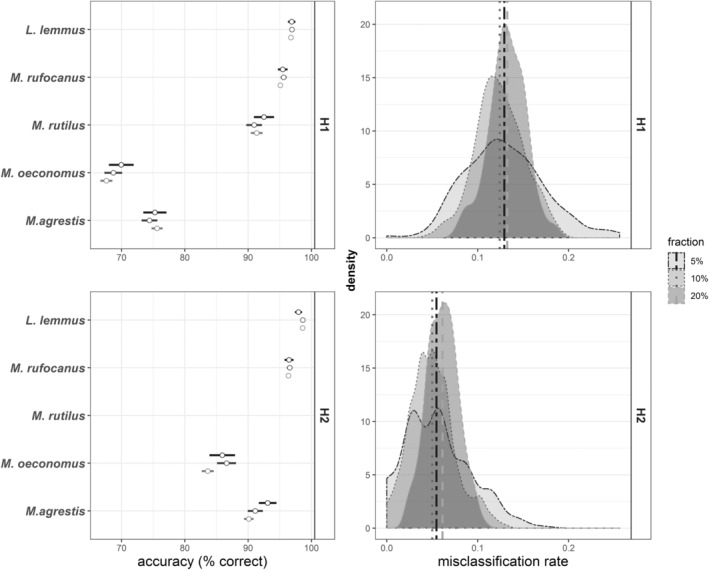
Predictive ability of species‐specific fNIRS calibrations for small rodents from Finnmark, Norway. **Left panels:** prediction accuracy of each species (mean ± sd) separately. **Right panels:** density plots of model misclassification rates. **Upper row** shows results from calibration models used for H1 (i.e., including all data from West Finnmark), **lower row** for H2 (i.e., including only samples used to test exposure effect). Results are divided by data splits (fractions) with 200 iterations each, where 5%, 10%, and 20% of data were randomly assigned as validation data (using rdMCCV framework). *M. rutilus* samples were not included in exposure treatment and H2 model.

Calibration models for genus identity resulted in excellent prediction accuracy and a low misclassification rate of 0.041 ± 0.019 (mean ± sd, Figure 2, upper right panel). Across all model iterations, prediction accuracy for *Myodes* was 96.2% ± 3.13%, for *Microtus* 95.1% ± 3.62% and for *Lemmus* 96.7 ± 3.47% (Figure 2, upper left panel). For species, the prediction accuracy was more variable and with a misclassification rate of 0.129 ± 0.032 (Figure 3, upper right panel). Prediction accuracies were high for *Myodes rufocanus* (95.4% ± 4.02%), *Myodes rutilus* (91.6% ± 9.20%) as well as *Lemmus lemmus* (96.1% ± 3.18%), but much poorer for *Microtus oeconomus* (68.8% ± 10.7%) and *Microtus agrestis* (75.1% ± 9.85%; Figure 3 upper left panel).

### 3.2 H2: Effect of feces exposure

Calibration models based on only exposed samples performed better than calibration models including intestinal samples, especially in predicting species identity (Figure 3). Effect of feces exposure to ambient weather for 1–6 weeks was visible on fNIR spectra, with a clear decline in reflectance at 700–1400 nm and increase in reflectance between ca. 1500 and 2500 nm (Figure S1). The rate of change in fNIR spectra seemed to decline with exposure duration for all species; however, the *Microtus oeconomus* and *M. agrestis* spectra changed strongly again after week 5 (Supplementary material S1, Figure S1). Model misclassification rate for genus identity was 0.031 ± 0.02 (Figure 2, lower right panel), while mean prediction accuracy for *Myodes* was 96.7% ± 3.89%, for *Microtus* 95.7% ± 4.24% and for *Lemmus* 98.3 ± 2.86% (Figure 2, lower left panel). Model misclassification rates for species calibrations were low, with 0.055 ± 0.028, and mean prediction accuracies reached 96.5 ± 3.76% for *Myodes rufocanus*, 98.4% ± 2.79% for *Lemmus lemmus* and were as high as 85.4% ± 11.2% for *Microtus oeconomus* and 91.4% ± 8.25% for *Microtus agrestis* (Figure 3, lower row).

**FIGURE 4:**
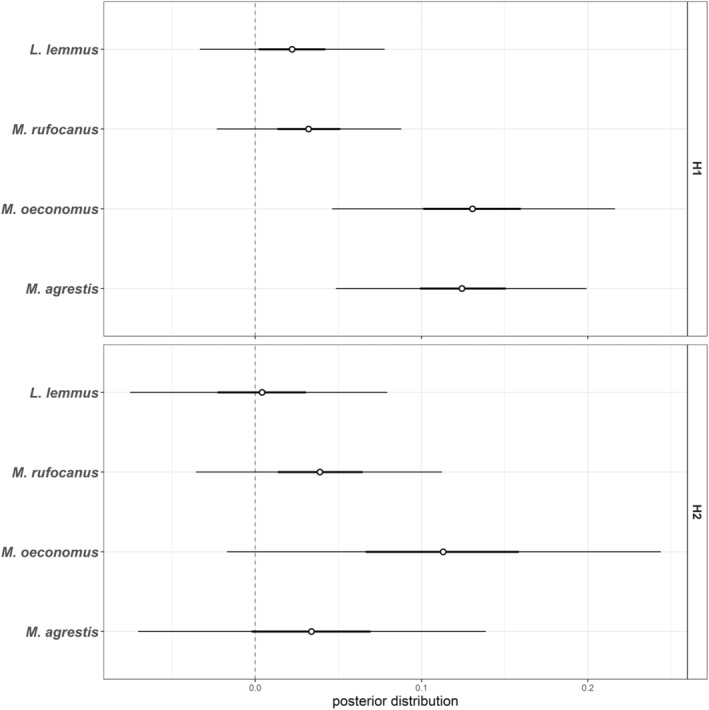
Beta‐regression posterior distributions (mean ± 95% CI) of exposure duration (in weeks) effect on individual prediction accuracy (IPA) of four species included in exposure treatment. Upper panel shows model results where IPA values are based on H1 calibration, whereas in lower panel IPA values are based on H2 calibration.

Posterior distributions of exposure week effects on IPA values indicated a positive effect of exposure on *Microtus oeconomus* and *M. agrestis* prediction accuracy when both intestinal and exposed samples were included in calibration (H1 model). However, exposure did not affect IPA values when only the exposed samples we used in calibration (H2 model). Exposure treatment did not affect individual prediction accuracies of other species with 95% confidence limits (Figure 4, Table S3).

### 3.3 H3: Does diet confound prediction accuracy?

The fNIR spectra were only weakly correlated to diet, as indicated by both Mantel test (*r* = .153, *p* < .001) and Procrustes test (m12‐squared = 0.932, correlation = 0.261, *p* < .001).

**FIGURE 5:**
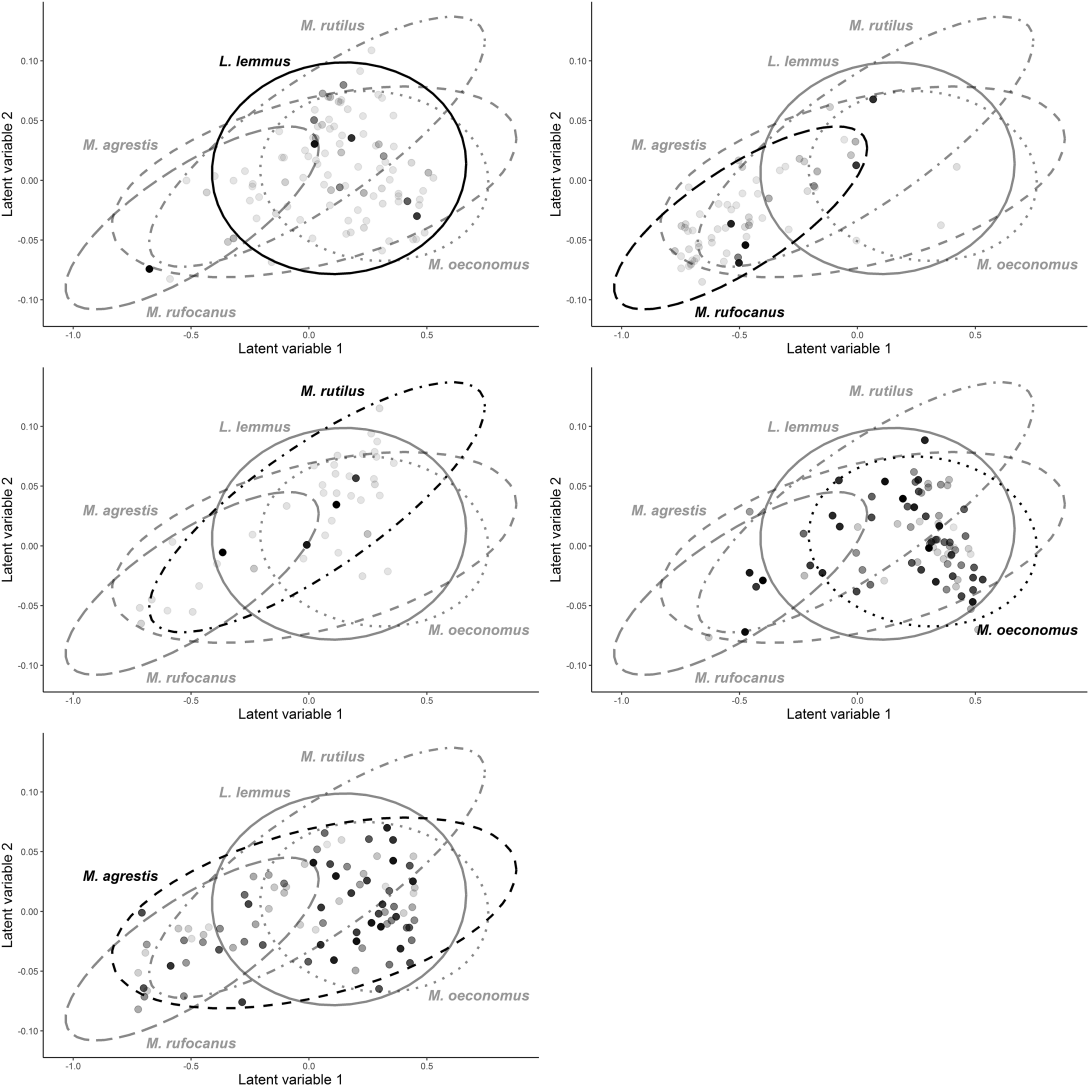
Comparing variation in diet composition with individual sample misclassification rate. Each individual (sample) is plotted as a separate dot, and all five species are plotted in their separate subplots. Spreads of species‐specific diets are shown by separate ellipses. Point color darkness indicates lower IPA value, that is, higher sample misclassification rate based on rdMCCV calibration models on plotted samples. Point locations are latent variables of relative read abundances. Latent variable modeling is equivalent to unconstrained ordination, that is, proximity of samples in two‐dimensional space indicates diet similarity, and distance indicates dissimilarity.

Latent variable modeling indicated clear overlap between most species' diets. However, *Myodes rufocanus* and *M. rutilus* were clearly differentiated (Figure 5). Most of the individuals with low IPA values (i.e., individuals that were often classified to wrong species) were located among the dense diet cluster of their respective species, while many peripheral individuals in terms of diet had high IPA scores (Figure 5).

### 3.4 H4: Regional vs. cross‐regional calibrations

**FIGURE 6:**
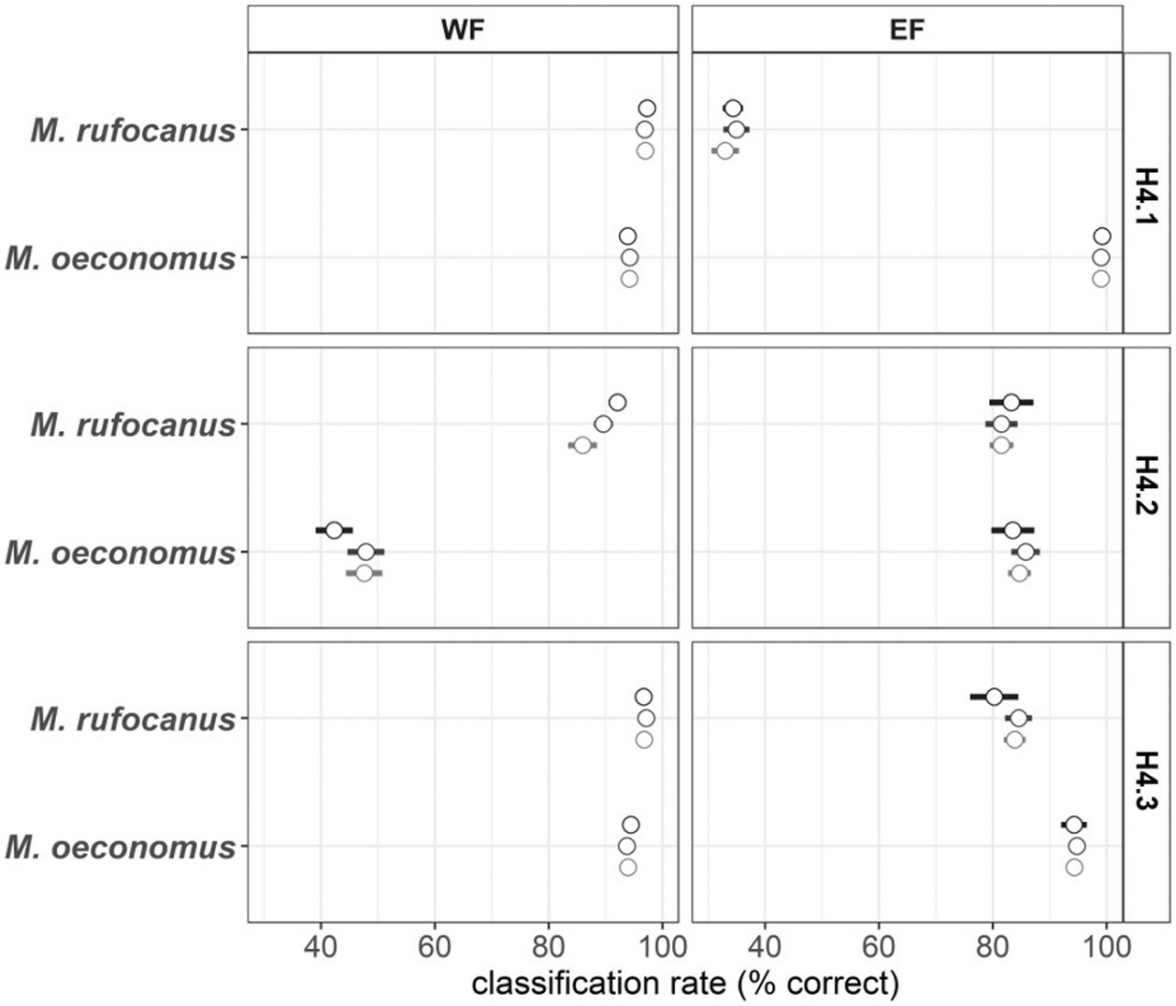
H4 model prediction accuracy (mean ± sd) for WF and EF separately. Upper row: H4.1 calibration is based on WF data only. Middle row: H4.2 calibration is based on EF data only. Lower row: H4.3, calibration is based on jointly WF and EF data.

Calibration models built with data from one region predicted samples from the same region well, but samples from the other region poorly (Figure 6; cf. Box 1 and Figure 8). WF calibration (H4.1) predicted WF samples with a misclassification rate of 0.041 ± 0.027, while misclassification rate for EF samples was as high as 0.349 ± 0.071 (Supplementary material S1, Figure S2). Mean prediction accuracy of *Myodes rufocanus* was 96.8% ± 3.14% in WF samples, but only 27.1% ± 15.6 in EF samples, while prediction accuracy of *Microtus oeconomus* samples from WF was high (94.2% ± 5.30%), but not as high as in EF samples (99.4% ± 2.70%; Figure 6 upper row).

BOX 1 **Practical application of the calibration model**

**Step 1: Building the calibration model.**

We used 95% of exposed WF samples (*n* = 798, modeling dataset) to fit an FDA/MARS model with a two‐way interaction. In total, 66 wavelengths were selected into the model (Figure 9 lower panel, Supplementary material S1, Figure S3).

**Step 2: Predicting new samples.**

We used the calibration model to predict the remaining 5% of WF {*n* = 35), and all EF samples to species. Misclassification rate of the WF samples was zero, that is, their prediction accuracy was 100% for all four species, *Myodes rufocanus, Microtus oeconomus* and *Microtus agrestis*, and *Lemmus lemmus* (cf. Figure 3 lower right panel). The misclassification rate of EF samples was 0.875, with prediction accuracies of 13.2% for *M. rufocanus* and 11.2% for *M. oeconomus*.

**Step 3: Visualizing the calibration model and sample misclassification.**

**FIGURE 7:**
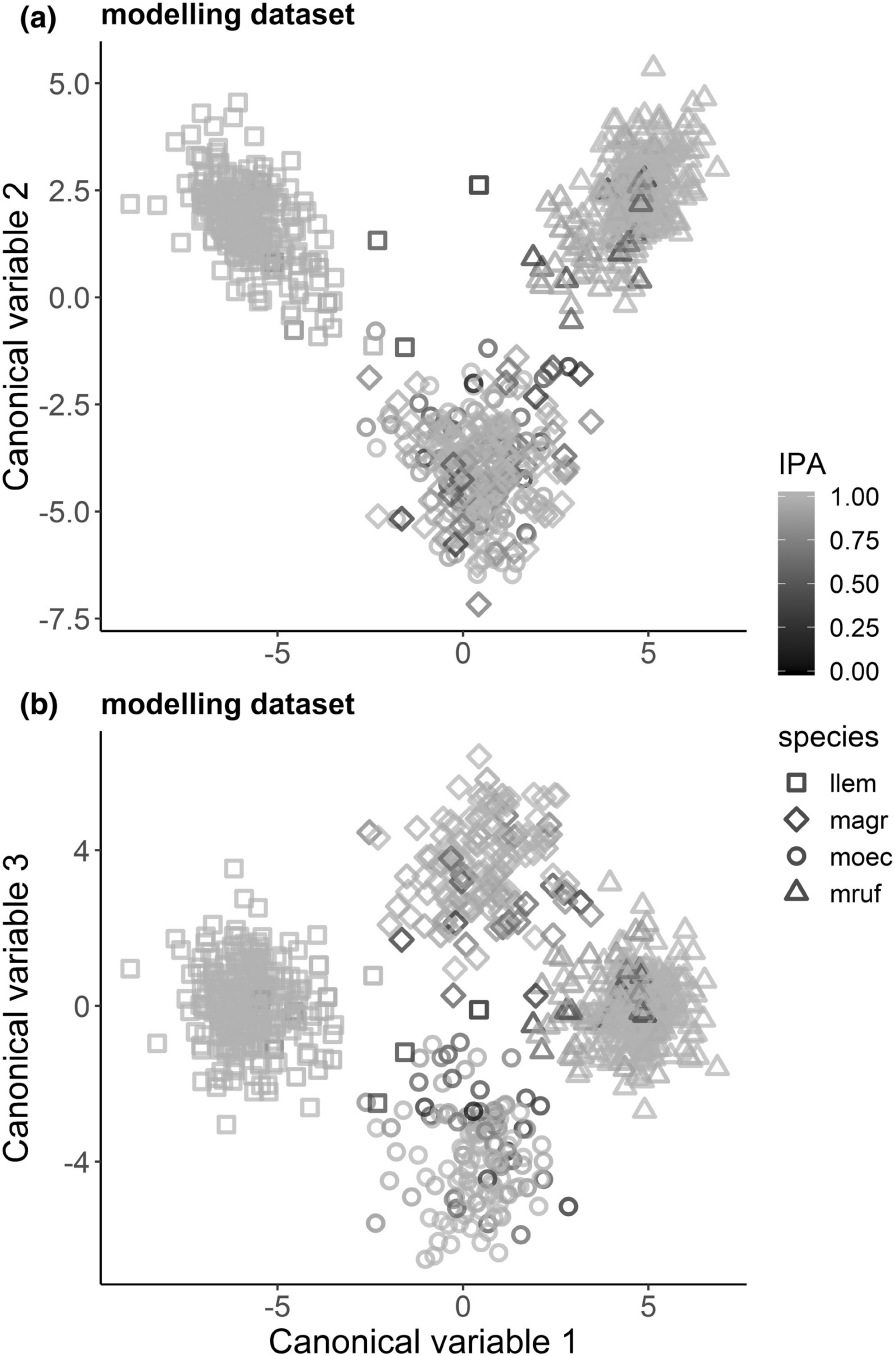
Canonical discriminant plot of the calibration model. Each point indicates modeling dataset sample position in the FDA model's 3D canonical discriminant space. Point shape indicates species identity, and point shade of gray indicates the individual sample IPA value so that hue darkness denotes increasing misclassification rate. llem, *Lemmus lemmus*; magr, *Microtus agrestis*; moec, *Microtus oeconomus*; mruf, *Myodes rufocanus*.

**FIGURE 8:**
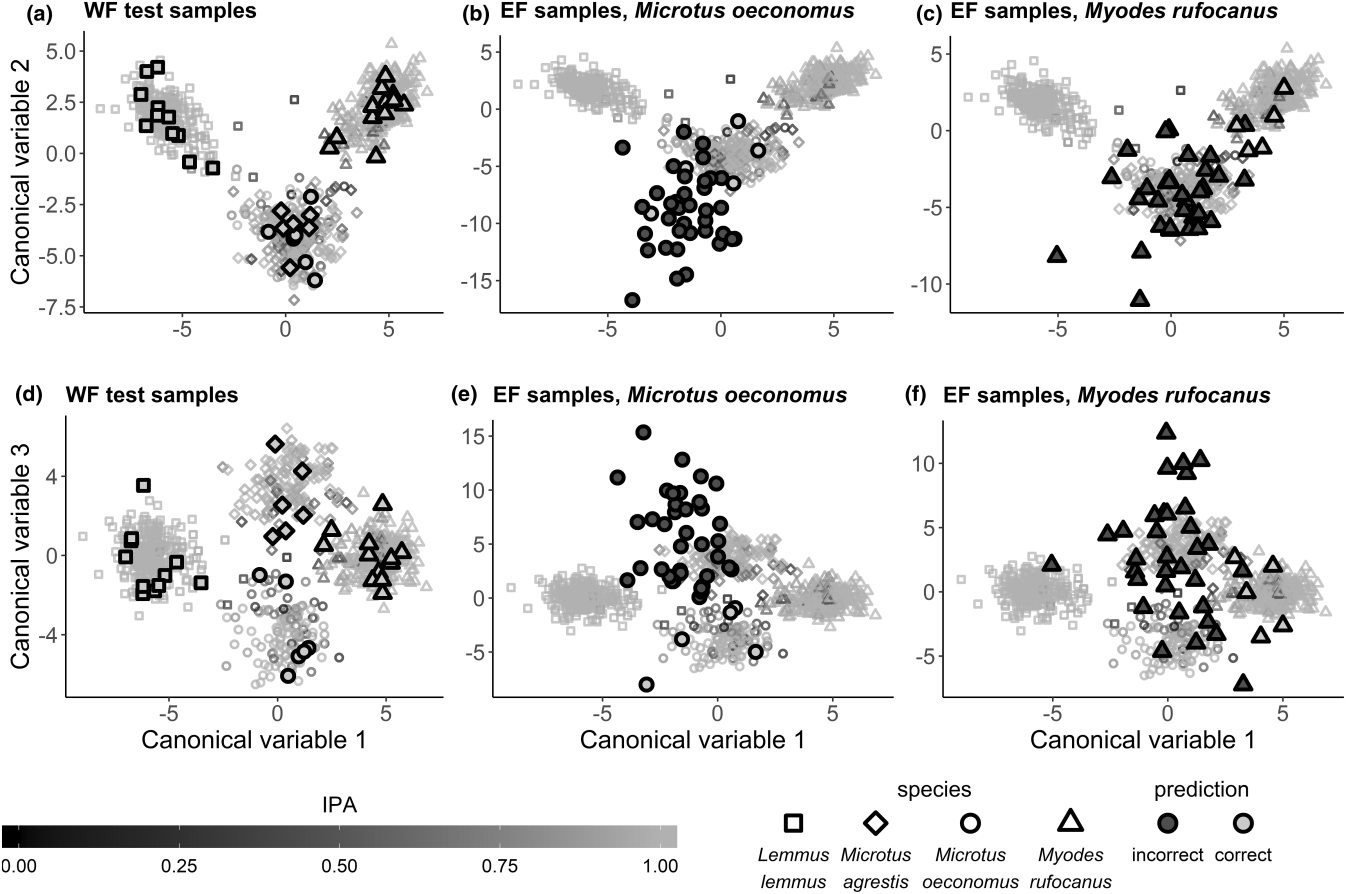
Canonical discriminant plot of the calibration model, with predicted locations of WF and EF test samples plotted on top of the modeling dataset points (cf. Figure 7). Point shape indicates species identity. WF and EF samples are plotted with large points with black borders and with fill color indicating correct or incorrect calibration model prediction. Modeling data are plotted in all panels as small transparent gray points as in Figure 7.

**FIGURE 9:**
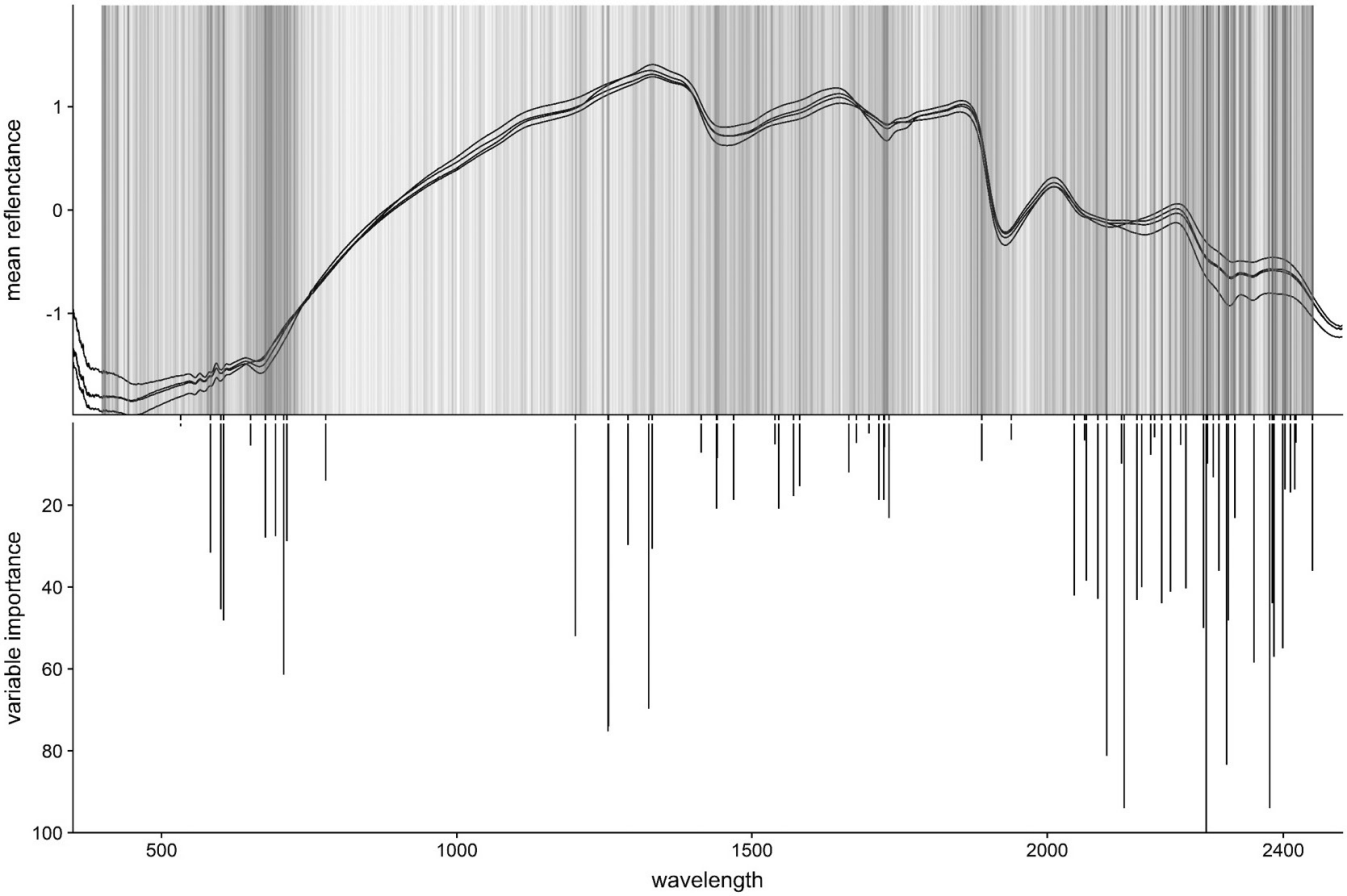
Comparison of the calibration model's variable selection and variable importance (VI) with the variable selection across all rdMCCV models. Wavelength is plotted on the x‐axis. **Upper panel:** Wavelengths with VI > 0 across all rdMCCV iterations. Increasing hue darkness indicates higher frequency of VI > 0 for that wavelength across all 600 rdMCCV iterations (a gray, transparent vertical line is plotted each time a wavelength receives a VI score > 0). Plot includes mean spectra of all four species in the calibration model, to visualize differences in spectra with important wavelength distribution. **Lower panel:** Wavelengths with VI > 0 in the calibration model, with VI score on the reversed y‐axis.

First, we plotted the canonical discriminant variables (CA1‐3) of the modeling dataset (Figure 7) and the WF and EF test sets (Figure 8). Second, to illustrate areas of high and low sample misclassification rates in the discriminant space, we applied the rdMCCV procedure on the modeling dataset and retrieved sample IPA values to color the plotted samples (Figures 7 and 8). Misclassification rate of the modeling data based on the rdMCCV calibration models was 0.056 ± 0.028. Across the 600 rdMCCV models, in total 1961 variables had importance greater than zero in one or more models (Figure 9 upper panel, Figure S3).

Canonical discriminant space of the calibration model showed a clear separation of *Lemmus lemmus*, *Myodes rufocanus* and *Microtus* along CA1 and CA2 (Figure 7a), whereas *Microtus oeconomus* and *Microtus agrestis* separated along the thud canonical variable CA3 (Figure 7b). Most, but not all, individuals with high misclassification rates (low IPA values) were located in peripheral areas of the respective species clusters, that is, toward the central region of the plot (Figure 7).

Figure 8 visualizes how the calibration model predicted WF samples within and EF samples outside the modeling data population, illustrative of model results for H4. The WF test set samples discriminated within then respective species clusters with high IPA values of modeling dataset samples (Figure 8a,b) and were predicted accurately. The poorly predicted EF test set samples (*M. oeconomus* and *M. rufocanus*) positioned both outside the calibration data range and close to *M. agrestis* modeling dataset samples (Figure 8c,d and Figure 8e,f, respectively).

Similar region‐specific pattern emerged with calibration models built on EF data (H4.2). Misclassification rate was lower for EF (0.171 ± 0.140) than for WF samples (0.265 ± 0.064, Figure S2). Prediction accuracy for *M. oeconomus* form WF was especially poor (47.9% ± 20.2%), while *M. rufocanus* reached higher prediction accuracy (89.7% ± 12.8%; Figure 6 middle row). By contrast, both *M. oeconomus* (84.9% ± 19.6%) and *M. rufocanus* (80.8% ± 21.4%) from EF test data reached decent prediction accuracies (Figure 6 middle row).

Calibration model including both WF and EF samples (H4.3) predicted test samples nearly as well or better than either of the regional models alone (Figure 6). Misclassification rate for WF samples was 0.041 ± 0.027, and for EF samples, 0.104 ± 0.108 (Figure S2). Mean prediction accuracy for *M. rufocanus* was 96.7% ± 3.25% for WF and 85.3% ± 18.5% for EF samples (Figure 6 lower row). Models predicted *M. oeconomus* at 94.6% ± 5.02% and 93.9% ± 13.2% in WF and EF samples, respectively (Figure 6 lower row).

## 4 DISCUSSION

Our results demonstrate fNIRS as an accurate method for predicting vole and lemming genus and species identity based on single fecal pellets (H1 and H2). While the calibration models predicted genus identity extremely well (at >95% accuracy), prediction accuracy of species identity varied more but was still good‐to‐excellent at 85%–98%. Exposure of samples to ambient weather resulted in the best calibration model performance, contradicting the hypothesized negative effect of collecting samples from the field (H1 vs. H2). Surprisingly, we did not find support for diet being a major determinant of species prediction accuracy (H3). Calibration models including samples from two regions provide good prediction accuracy for both regions, corroborating our fourth hypothesis (H4.3). However, calibration models based on samples from one region may not readily be applicable to samples from another region (H4.1, H4.2). Our findings are in line with previous results of reaching good prediction accuracies when calibration datasets are expanded in space or time (Murguzur et al., 2019; Tolleson et al., 2005).

Our results lend strong support for using fNIRS on samples exposed to ambient conditions, that is, samples collected from the field, and for further development of fNIRS for large‐scale rodent population monitoring, including cross‐regional calibrations. For future applications, the biggest appeal lies in the versatility of fNIRS. In addition to small rodent censuses or community sampling, fNIRS calibrations can provide information on demography, fecal nutrients, stress hormones, and even disease, variables linked with fitness and not all captured by genetic methods—all from the same NIR spectra. This makes fNIRS a compelling addition to ecosystem monitoring and sampling toolkits.

### 4.1 Diet and misclassification

Dried feces consist of ingested diet material, gut microbes and sloughed tissue, gastric secretions, and metabolized hormones, of which all may carry species‐specific chemical or structural imprints. Of these, we explicitly addressed the link between diet composition and misclassification. We did not find strong evidence of diet composition explaining individual sample misclassification. This result is surprising as diet quality measures such as fecal nitrogen, neutral and acid detergent fiber (NDF, ADF), crude fiber, lignin, ether extracts, and ash are well predictable from fNIR spectra (Gil‐Jiménez et al., 2015; Steyaert et al., 2012). If diet quality and composition are only loosely associated, the link between fNIR spectra, species identity, and generalist diet composition may not be easily detected. Even so, it is likely that group‐specific variation in diet composition affecting any diet quality variable would still affect misclassification rates (e.g., Tolleson et al., 2005). Further research is needed to determine whether and which diet composition or quality variables co‐vary with species identity to affect fNIRS rodent species classification.

Furthermore, the large sample size from WF may have incorporated sufficiently high levels of intraspecific dietary variation, to render the effect of diet on misclassification rate small, compared with other fecal constituents. With lower sample sizes, systematic variation in diet, for example, between years could confound species identification as found by Tolleson et al. (2005). Similarly, systematic variation in species diet between regions could explain high rates of misclassifications between WF and EF. Species‐specific diets between these regions may differ given different rodent species guild composition, habitat composition, and forage availability, which could translate to variation in dietary quality. With WF calibrations, both *M. rufocanus* and *M. oeconomus* from EF were frequently misclassified, as they appeared to cluster together with *M. agrestis*—a species absent from Eastern Finnmark. It is possible that in the absence of *M. agrestis*, which competes with both *M. oeconomus* and *M. rufocanus* at WF, these latter two species' dietary niches in EF resemble that of *M. agrestis* at WF.

### 4.2 Juxtaposing misclassification with phylogenetic history

In general, classification model performance emerges likely as a complex product of individual traits linked with phylogenetic history. Evolutionary constraints may manifest in fNIR spectra through morphological, physiological, or microbiological differences in the digestive system, along with ecological niche factors (Blomberg et al., 2003). Dental and gut morphologies are examples of phylogenetically variable traits that may affect fNIRS species identification through differences in fecal particle size (Clauss et al., 2015; Sheine & Kay, 1977) and fecal nitrogen levels or fiber digestibility (Clauss et al., 2015; Lovegrove, 2010), respectively (Foley et al., 1998; Tolleson et al., 2005 and references therein; Steyaert et al., 2012).

Indeed, divergence of *Lemmus*, *Myodes,* and *Microtus* at tribal level (Buzan et al., 2008) is congruent with good performance of calibration models at genus level. Similarly, *Myodes rufocanus* and *M. rutilus* which were well separated in classification models show marked phylogenetic differentiation within the genus (Buzan et al., 2008; Cook et al., 2004; Kohli et al., 2014). While the two *Myodes* species' ecology can be similar in, for example, subarctic birch forests (cf. Ehrich et al., 2009), clear behavioral niche differences may exist (Nations & Olson, 2015) and for instance their habitat use at JRA differs markedly. By contrast, higher misclassification rates and similarities in *Microtus* fNIR spectra link with the relatively recent and rapid radiation history of the genus (Barbosa et al., 2018). While *M. oeconomus* is ancestral to *M. agrestis*, there is ongoing intraspecific divergence within both species (Barbosa et al., 2018), as well as strong interspecific competition (e.g., Hoset & Steen, 2007). Divergence patterns in dental structure converge roughly with these phylogenetic patterns, with *Microtus oeconomus* and *M. agrestis* displaying the smallest differences (Herrmann, 2002).

### 4.3 Identification of species and pellet exposure

Surprisingly, misclassification rates of our samples decreased after exposure to ambient weather. This was mainly due to higher prediction accuracy within the genus *Microtus*, when we excluded intestinal samples from modeling. It appears that species‐specific signals in fNIR spectra of the closely related *M. oeconomus* and *M. agrestis* became more apparent with exposure. While we found no evidence of reduced prediction accuracy with increasing exposure time, rates of visible changes in spectra varied between weeks and species. Yet, it is likely that exposure times longer than 6 weeks will eventually lead to loss of species‐specific signals as the fecal pellets decompose. Further research is needed to determine the maximum or optimal timeframe for successful classification of field‐sampled rodent feces. How this compares with degradation of genetic material might indicate the suitability of genetic vs. spectral analysis for old fecal samples.

Comparing models that include both intestinal and exposed samples to models with exposed samples only allows for speculation as to which fecal constituents may have contributed to species identification, based on constituents' known susceptibility to exposure. Specifically, species‐specific signals in fNIR spectra seem not to associate with volatile or rapidly leaching substances. For instance, stability of constituents linked with dietary quality in fecal samples varies strongly under exposure (Jenks et al., 1990; Kamler et al., 2003; Leite & Stuth, 1994; Steyaert et al., 2012). Gut microbiota and the fecal metabolome likely displays phylogenetic and species‐specific variation (Anders et al., 2021; Zierer et al., 2018) detectable by fNIRS (Santos et al., 2014; Saric et al., 2008). Fecal steroid metabolite concentrations of various large mammal species decline during days or remain stable for only up to a week (Abáigar et al., 2010; Mesa‐Cruz et al., 2014; Parnell et al., 2015). Thus, steroid metabolites may be too short‐lived to have accounted for species identification in our study. By contrast, differences in microbial flora could translate to some exposure‐resistant differences in rodent fNIR spectra. For instance, diaminopimelic acid (DAPA), a marker of gut microbe‐derived N in feces (Karr‐Lilienthal et al., 2004), has good NIRS calibrations (Atanassova et al., 1998) and has been found to retain stable concentrations in exposed deer feces (Kamler et al., 2003). In summary, rodent species identification is likely to rest on a multitude of fecal constituents, many of which link with phylogenetic distance (Ley et al., 2008; Zierer et al., 2018) and have varying resistance to exposure and decay. We note that quoted studies on exposure effects on fecal dietary quality indicators and on fecal metabolome cover only up to a few weeks or days, respectively, providing only a preliminary basis for interpretation of constituents affecting fNIRS calibrations.

### 4.4 Toward application of fNIRS calibrations for rodent monitoring

Our results corroborate previous findings of breaking past limitations of closed sample populations (Murguzur et al., 2019), as we show that increasing the spatial extent of calibration data will produce robust models across sample populations. However, we also caution for high or increased misclassification rates of samples from ecological contexts outside the calibration range (Tolleson et al., 2005; Murguzur et al., 2019) and advise occasional validation of few predicted samples, as well as inclusion of feces from contrasting biomes, seasons, and populations. New samples from external populations may exhibit considerably different variation in constituents underlying the species‐specific signal compared with calibration samples, making their predictive modeling highly risky. This is hardly surprising, if the species‐specific signal is co‐determined by a complex host of constituents, some of which are linked to community‐specific interactions (e.g., diet, disease, and body condition), while others would be more unequivocally associated with phylogenetic constraints (gut or dental morphology and gut microbiome). Therefore, increasing the variability in calibration data should mask constituents linked with community‐specific interactions and lead to the detection of wavelength combinations predictive of species identity across populations. While diet, habitat use and gut morphology of microtine rodents show high levels of plasticity (Lovegrove, 2010; Soininen et al., 2013), a growing body of fNIRS research (Aw & Ballard, 2019; Tolleson, 2010; Vance et al., 2016) suggests that robust species‐specific markers are also likely to exist.

Practical application of fNIRS for monitoring and census purposes rests on good understanding of model behavior and limitations of calibration models. Notably, we found that the selected wavelengths varied considerably between model iterations (see Box 1), thus providing little support for any definite set of wavelengths being superior for species identification. Instead, our modeling framework provides a visual tool to assess misclassification risk of new samples derived from the calibration data population (Figures 7 and 8). Samples located well within species clusters in the discriminant space are likely to be predicted correctly, while samples falling between the clusters have a considerably higher risk of misclassification. Such samples should optimally be used to improve model decision boundaries by including them in the model calibration. To control for interannual variation or drift in, for example, diet in long‐term monitoring, a small subset of new samples should, at regular intervals if possible, undergo an external verification of species identity, and thus compliment the calibration dataset.

In conclusion, fNIRS can facilitate rodent population censuses with larger sample sizes, combining large spatial extent with small grain if combined with pellet‐count‐based abundance indices (Engeman & Whisson, 2006; Jareño et al., 2014; Karels et al., 2004). The wide array of data (e.g., diet, disease, and stress) discernible through fNIRS suggests that developing monitoring schemes based on pellet counts and fNIRS could meet the need for ecosystem‐ and interaction‐based approaches to monitoring (Ehrich et al., 2019) and for this purpose complement genetic methods (Zemanova, 2021). Further steps in this direction involve increasing the spatial scope of calibrations, extending the calibration exposure times to meet the needs of field sampling intervals, as well as continuous development of the calibration model algorithm and outlier detection. Key advantage of fNIRS is the ability to use existing spectral data to develop calibrations for any number of qualitative and quantitative variables (Foley et al., 1998; Vance et al., 2016), opening possibilities to link process and pattern across different levels of organization. For small rodents, fNIRS could provide invaluable data for nutritional ecology, stress and disease, underlying population dynamics and biotic interactions. Thus, given the emergence of active global researcher networks (e.g., Barrio et al., 2016) and spectral libraries (e.g., Shepherd & Walsh, 2002), we propose initiatives toward development of circumpolar, −boreal or global rodent fNIRS calibrations.

## AUTHOR CONTRIBUTIONS

**Maria W. Tuomi:** Conceptualization (equal); data curation (lead); formal analysis (lead); funding acquisition (supporting); investigation (equal); methodology (lead); project administration (lead); resources (equal); software (lead); validation (lead); Visualization (Lead), writing – original draft preparation (lead); writing – review and editing (lead). **Francisco J. A. Murguzur:** Conceptualization (equal); data curation (supporting); formal analysis (equal); investigation (lead); methodology (equal); resources (equal); software (equal); validation (supporting); writing – review and editing (equal). **Katrine S. Hoset:** Funding acquisition (equal); methodology (supporting); project administration (equal); resources (equal); writing – review and editing (equal). **Eeva M. Soininen:** Formal analysis (supporting); methodology (supporting); resources (equal); writing – original draft (supporting); writing – review and editing (equal). **Eero J. Vesterinen:** Data curation (supporting); formal analysis (equal); methodology (supporting); resources (equal); software (supporting); writing – review and editing (equal). **Sissel Kaino:** Investigation (supporting); resources (equal); writing – review and editing (supporting). **Tove Aagnes Utsi:** Resources (equal); writing – review and editing (equal). **Kari Anne Bråthen:** Conceptualization (equal); funding acquisition (lead); project administration (equal); resources (lead); supervision (lead); writing – review and editing (equal).

## CONFLICT OF INTEREST STATEMENT

The authors declare no competing interests.

### OPEN RESEARCH BADGES

This article has earned an Open Data badge for making publicly available the digitally‐shareable data necessary to reproduce the reported results. The data is available at [[insert provided URL from Open Research Disclosure Form]].

## Supporting information


Appendix S1.

## Data Availability

Data and code to replicate all analysis in this study (small rodent fNIR‐spectra, dietary data with masked species identities and R scripts) are openly available at UiT Open Research Data at https://doi.org/10.18710/9QKUIQ.
